# Increased incidence of precocious and accelerated puberty in females during and after the Italian lockdown for the coronavirus 2019 (COVID-19) pandemic

**DOI:** 10.1186/s13052-020-00931-3

**Published:** 2020-11-04

**Authors:** Stefano Stagi, Salvatore De Masi, Erica Bencini, Stefania Losi, Silvia Paci, Maria Parpagnoli, Franco Ricci, Daniele Ciofi, Chiara Azzari

**Affiliations:** 1 Health Sciences Department, Anna Meyer Children’s University Hospital, University of Florence, Viale Pieraccini 24, 50139 Florence, Italy; 2 Anna Meyer Children’s University Hospital, Viale Pieraccini 24, 50139, Florence, Italy

**Keywords:** Precocious puberty, Accelerated puberty, COVID-19

## Abstract

**Background:**

The timing of puberty in girls is occurring at an increasingly early age. While a positive family history is recognised as a predisposing factor for early or precocious puberty, the role of environmental factors is not fully understood.

**Aims of the study:**

To make a retrospective evaluation of the incidence of newly diagnosed central precocious puberty (CPP) and the rate of pubertal progression in previously diagnosed patients during and after the Italian lockdown for COVID-19, comparing data with corresponding data from the previous 5 years. To determine whether body mass index (BMI) and the use of electronic devices increased during lockdown in these patients.

**Patients and methods:**

The study included 49 females with CPP. We divided the patients into two groups: group 1, patients presenting a newly diagnosed CPP and group 2, patients with previously diagnosed slow progression CPP whose pubertal progression accelerated during or after lockdown. We collected auxological, clinical, endocrinological and radiological data which were compared with data from two corresponding control groups (patients followed by our Unit, March to July 2015–2019). Patients’ families completed a questionnaire to assess differences in the use of electronic devices before and during lockdown.

**Results:**

Thirty-seven patients presented newly diagnosed CPP (group 1) and 12, with previously diagnosed but untreated slow progression CPP presented an acceleration in the rate of pubertal progression (group 2). The number of new CPP diagnoses was significantly higher than the mean for the same period of the previous 5 years (*p* < 0.0005). There were no significant differences between patients in group 1 and control group 1 regarding time between appearance of B2 and CPP diagnosis, although group 1 patients had a significantly earlier chronological age at B2, a more advanced Tanner stage at diagnosis (*p* < 0.005), higher basal LH and E2 levels, higher LH peak after LHRH test (*p* < 0.05) and increased uterine length (*p* < 0.005) and ovarian volume (*p* < 0.0005). The number of patients with previously diagnosed CPP whose pubertal development accelerated was also statistically higher compared to controls (*p* < 0.0005). In this group, patients’ basal LH (*p* < 0.05) and E2 levels (*p* < 0.0005) became more markedly elevated as did the LH peak after LHRH test (*p* < 0.05). These patients also showed a significantly accelerated progression rate as measured by the Tanner scale (*p* < 0.0005), uterine length (*p* < 0.005), and ovarian volume (*p* < 0.0005). In both group 1 and group 2, BMI increased significantly (*p* < 0.05) and patients’ families reported an increased use of electronic devices (*p* < 0.0005).

**Conclusion:**

Our data show an increased incidence of newly diagnosed CPP and a faster rate of pubertal progression in patients with a previous diagnosis, during and after lockdown compared to previous years. We hypothesize that triggering environmental factors, such as the BMI and the use of electronic devices, were enhanced during lockdown, stressing their possible role in triggering/influencing puberty and its progression. However, more studies are needed to determine which factors were involved and how they interacted.

## Introduction

Puberty is the result of a complex neuroendocrine system, initiated by an unknown primary mechanism, and characterized by the increased release of gonadotropin-releasing hormone (GnRH) by the hypothalamus [1, 2]. The phenomenon is related to a complex sequence of endocrine changes and is influenced by integrated multiple peripheral and central signals leading to the development of sex characteristics, the growth spurt and the obtaining of reproductive competence [1, 2].

Currently, abnormally precocious sexual development (precocious puberty or PP) is defined as the occurrence of Tanner stage B2 before 8 years in girls and Tanner stage G2 before 9 years in boys. The average age of onset of puberty has decreased over the last century [3–5]. Historical data from Europe show a sharp decline in mean age at menarche from approximately 17 years in the early nineteenth century to approximately 13 years by the mid-twentieth century, with a minor decrement during the past 25–30 years [5]. Mean age at onset of breast development has also decreased in all ethnic groups [6], as reported by the Pediatric Research in Office Settings (PROS) study [3] and the data by Biro et al. [7]. These same data show a progressive increase in the number of girls fulfilling the criteria for PP (6.7% in the PROS study and 10.4% in the Biro data for white girls) [6, 7].

Genetic factors undoubtedly play a major role in these trends [8]. However, there is a consensus that environmental variables, such as weight, foetal nutrition, childhood dietary habits, physical activity, psychological factors, and exposure to electromagnetic fields (EMF) and/or endocrine disrupting chemicals, also have an impact [2, 5, 9].

In Italy, as elsewhere, the COVID-19 pandemic created an unprecedented strain on health care and during the strictest months of lockdown forced millions of people to remain isolated in their homes for about 2 months, from March to May 2020 [10, 11]. It is possible that the lockdown enhanced the impact of factors interfering with the timing and *tempo* of puberty, such as adiposity [12, 13] and psychological wellbeing. An increased incidence of depression, anxiety and stress have been reported to varying degrees in adults [14], and even if comprehensive data among children and adolescents are scarce, it is likely that children had similar psychological reactions.

The aims of this study are to evaluate the frequency of precocious puberty and the rate of pubertal progression in girls during and after the lockdown, comparing results with data for the same period over the last 5 years and to consider possible correlations between lockdown conditions and trends in the data.

## Patients and methods

We retrospectively evaluated the medical records of Caucasian patients referred to the Auxoendocrinology and Paediatric Gynaecology Unit of the Meyer Children’s University Hospital for CPP (group 1) during and after the Italian lockdown for the COVID-19 infection (March – July 2020), comparing this data with the medical records of the same period of the previous 5 years (March–July 2015–2019) (control group 1). We also collected data on the rate of pubertal progression (the *tempo* of puberty) in patients already followed by our Unit for untreated and slow progressive PP (group 2), again comparing the data with the same period of the previous 5 years (control group 2).

Patients with a diagnosis of CPP and associated hypothalamic–pituitary congenital malformations, neurological, neurosurgical and/or genetic diseases, psychomotor delay, oncological diseases, other endocrine impairments requiring hormonal treatments, or taking drugs that may interfere with pubertal development were excluded from the study. We also excluded children who were adopted or had immigrated to Italy as such children have a statistically higher rate of precocious puberty than the general pediatric population.

The study was performed according to the Helsinki II declaration and approved by the local Paediatric Ethical Committee (approval number: 30/07/2020–179). Written informed consent was obtained from the parents of enrolled children.

### Study design

All patients underwent a physical examination and auxological evaluation. We recorded height, weight, body mass index (BMI), height velocity (HV) and stage and rate of pubertal progression (Tanner scale) [15]. HV was calculated at least once in every year and was defined as the increase in height in centimeters per year [15]. BMI was calculated by dividing the patient’s weight in kilograms by the square of height in meters [16]. Height, HV and BMI were normalized for chronological age by conversion to SD scores [15]. When possible, target height (TH) SD scores were also calculated.

If available, we collected clinical data (e.g. age at CPP diagnosis, personal and family history for major diseases, family history for CPP) and endocrinological data (follicle-stimulating hormone [FSH], luteinizing hormone [LH], estradiol [E2], dehydroepiandrosterone sulfate [DHEAS], 17-hydroxyprogesterone [17-OHP], prolactin [PRL], free thyroxine [FT4], thyroid-stimulating hormone [TSH]). In addition, a pelvic ultrasonography, a bone age (BA) assessment using radiographs of the left hand and wrist and a LH-releasing hormone (LHRH) stimulation test were performed.

Pubertal development was classified according to the Marshall and Tanner criteria [17]. The age of pubertal onset was defined as the age at durable Tanner B2 stage, confirmed by the auxological, endocrinological and/or radiological results [14]. The rate of pubertal progression was taken to be the time from B2 to B3 and/or B4 [14].

Skeletal maturation was expressed as BA and evaluated, when possible, as the ratio of the change in BA to the change in chronological age (CA) (BA/CA), and as the difference between CA and BA (CA minus BA) in years [14].

Precocious puberty was defined as the development of pubertal changes at an age that was younger than the accepted lower limits for age of onset of puberty (before the age of 8 years in girls) [18]. We considered peak LH values of > 5 IU/L on the GnRH in the presence of pubertal signs or a basal LH value of > 1.1 IU/L and a ratio of stimulated LH to stimulated FSH of > 1.0 combined with isolated and/or axillary hair growth accompanied by breast development [18] to be indicative of activation of the hypothalamic GnRH pulse generator.

For the purposes of this study, the rate of puberty progression was evaluated:

- for group 1 patients with new CPP diagnoses and control group 1, by the time between the appearance of a durable Tanner stage 2 and diagnosis. For these patients auxological parameters were recorded at diagnosis and compared with those recorded by the patient’s paediatrician at the moment of referral.
- for group 2 patients with untreated CPP patients who had presented slow progression prior to lockdown and control group 2, by the progression rate assessed by a Paediatric Endocrinologist (S.S.). All CPP patients are assessed every 3–4 months by our Unit to determine whether the rate of pubertal progression is stable or accelerating. We defined slow or accelerated pubertal development if the rate of progression from one pubertal stage to another is more or less than 6 months or 1 SD in comparison with the general population [19, 20].

Moreover, based on our previous data [21], we also evaluated quantitatively and qualitatively changes in the use of electronic devices before and during lockdown in these patients [21]. Parents were asked to complete a questionnaire specifying which devices children used, how long they used them for, at what times of the day and whether they were used by the children in their bedrooms during the hours before they went to sleep. The devices considered were: PCs, cell phones, MP3 players, tablets, game consoles and TVs. Data were collected after the lockdown. Time spent on screen-based activity was assessed by the following question: “including school hours (only during lockdown), how much time do you (the child) usually spend on the following: (1) TV, (2) TV-games (PlayStation, Xbox, WII, etc), (3) PC games, (4) Internet chatting, (5) using the PC for other purposes)?” The response alternatives were: ‘no time’, ‘less than ½ hour’, ‘½ hour to 1 hour’, 1 h to 2 h’, ‘2 hours to 3 hours’, ‘3 hours to 4 hours’, ‘4 hours to 5 hours’ and ‘more than 5 hours’. Most recommendations for screen-based activities restrict screen time for children and adolescents to about 2 h per day [22].

### Auxological evaluation

Height was measured using Harpenden’s stadiometer in triplicate to the nearest 0.1 cm. Weight was determined to the nearest 0.1 kg using a balance scale. Age-related reference values for height, bone age and BMI were those currently used in Italy, obtained in high sample numbers of Italian children, as previously reported [14, 15]. Height, HV, and BMI were normalized for chronological age by conversion to standard deviation (SD) scores according to the following formula: patient value – mean of age-related reference value/standard deviation of the age-related reference value [14, 15]. BA was evaluated through radiographs of the left hand and wrist and then calculated according to the Greulich and Pyle method [23]. TH was estimated according to the Hermanussen and Cole method [24], calculating mid-parent height as an SD score and correcting this by a factor corresponding to the influence of assortative mating and parent-offspring correlations.

### Pelvic ultrasonography

Pelvic ultrasonography was performed by the same operator (E.B.), using a Siemens Sonoline Elegra sonograph and a 6.5-MHz probe. The surface area (S) of the ovaries was calculated as follows: S = length x width × 0.8. The normal ovarian surface area is < 2 cm^2^ in prepubertal and from 2 to 6 cm^2^ in pubertal subjects [25].

### Laboratory methods

All laboratory measurements were performed on blood samples collected after overnight fasting. Plasma FSH and LH, PRL, 17OHP, DHEAS, FT4, TSH, and E2 levels were measured by means of chemiluminescent immunometric assays with the use of commercially available kits for the Immulite 2000 systems analyzer (Siemens Healthcare Diagnostics).

The LHRH test was carried out by taking blood samples at the 15th, 30th, 45th and 60th minute after intravenous administration of 100 mg/m2 (maximum 100 mg) LHRH (Lutrelef, 0.8 mg/10 mL; Ferring).

### Statistical analysis

Statistical analyses were performed with the use of SPSS X software (SPSSX Inc., Chicago, IL, USA). The characteristics of the study population were described using frequency distributions for categorical variables and mean and standard deviation (SD) values, medians, and ranges for continuous variables, depending on whether the data were normally distributed. The statistical significance of the continuous variable comparisons was assessed using the Student t test and the Mann-Whitney U test, depending on the distribution of the analyzed variable; the comparison of categorical variables was conducted using the chi square test or Fisher’s Exact test if there was a small (< 5) expected cell size. The Pearson correlation test was used to determine the correlation coefficients. All statistical tests were two-tailed and a *p* < 0.05 was considered statistically significant.

## Results

Clinical data and laboratory results were analysed in 49 females. All patients showed normal values of 17-OHP, TSH and FT4. Contrast enhanced brain MRI showed a normal hypophysis without focal abnormalities in all patients.

Thirty-seven patients (Group 1) received a new diagnosis of CPP during and after the Italian lockdown and 12 (Group 2), already diagnosed with slow progression CPP, presented an unexpected accelerated rate of pubertal progression in this period (Tables 1 and 2). The data of the new cases of CPP were compared with 89 patients with CPP diagnosed in the same period (March – July) of the years 2015–2019 (17.8 ± 1.3 patients, range from 16 to 19 cases/same period/year). The data of the 12 patients with slow progressive CPP in whom pubertal progression accelerated were compared with 11 patients with the same characteristics who had been followed by our Unit in the previous 5 years (2.2 ± 0.4 patients, ranging from 2 to 3 cases/same period/year).

**Table 1:** Clinical data and laboratory results in the patients of group 1 and controls

Variable		Group 1	Previous 5 years^**a**^	***P***
Population number		37	89	–
Chronological age at B2 (as referred by parents or family pediatrician)		6.86 ± 0.61	7.22 ± 0.48	*p* < 0.005
Chronological age at diagnosis (yr)		7.11 ± 0.72	7.53 ± 0.50	*p* < 0.0005
Time from B2 to diagnosis (months)		3.1 ± 0.9	3.0 ± 0.8	*P* = NS
Height, SDS		0.84 ± 1.32	0.79 ± 1.44	*P* = NS
BMI, SDS		0.83 ± 0.91	0.68 ± 0.88	*P* = NS
Tanner stage at diagnosis (percentage)				
II		43.8	55.5	
III		53,1	38.8	*p* < 0.05
IV		3.1	5.4	
V		–	–	
Bone age (yr)		9.40 ± 1.10	9.60 ± 1.20	*p* = NS
Bone age minus chronological age (yr)		2.29 ± 0.38	2.07 ± 0.70	*P* = NS
Basal LH, IU/L		1.2 ± 0.7	0.8 ± 0.6	*p* < 0.005
Basal FHS, IU/L		1.9 ± 1.7	2.2 ± 1.3	*p* = NS
Peak LH at GnRH stimulation, IU/L		11.9 ± 4.2	9.4 ± 4.0	*p* < 0.005
Basal estradiol (females only), pmol/L		129.9 ± 18.7	117.6 ± 19.2	*p* < 0.005
Uterine length, cm		4.42 ± 0.43	3.99 ± 0.47	*p* < 0.0005
Ovarian volume, cm^3^		3.32 ± 0.42	2.83 ± 0.46	*p* < 0.0005
Electronic device use (h)		3.9 ± 1.5	–	–

^a^= mean/yr. *BA* bone age, *CA* chronological age, *SDS* standard deviation score, *BMI* body mass index, *LH* luteinizing hormone, *FSH* follicle-stimulating hormone, *GnRH* gonadotropin releasing hormone test

**Table 2:** Clinical data and laboratory results of group 2 and controls (two evaluations after the same time of follow-up)

Variable	Group 2		Controls	
	before lockdown	after lockdown	visit 1	visit 2
Population number	12	12	11	11
Chronological age at diagnosis (yr)	7.47 ± 0.53	–	7.41 ± 0.61	–
Chronological age at follow-up (yr)	7.95 ± 0.49	8.26 ± 0.47	7.93 ± 0.51	8.31 ± 0.53
Time between the two visits (yr)	–	0.31 ± 0.02	–	0.38 ± 0.02
Height, SDS	0.87 ± 1.22	0.89 ± 1.25	0.86 ± 1.13	0.90 ± 1.16
BMI, SDS	0.61 ± 0.85	0.92 ± 0.87	0.60 ± 0.93	0.69 ± 0.94
Δ BMI, SDS	–	0.32 ± 0.02***	–	0.09 ± 0.01***
Tanner stage, percentage				
II	47.6	-***	63.6	54.5
III	52.4	71.4***	36.4	45.5
IV	–	28.6***	–	–
V	–	–	–	–
Basal LH (IU/L)	0.8 ± 0.6*	1.4 ± 0.6*	0.9 ± 0.7	1.1 ± 0.7
Basal FHS (IU/L)	1.9 ± 1.5	1.5 ± 1.3	1.6 ± 1.4	1.5 ± 1.2
Peak LH at GnRH stimulation (IU/L)	9.6 ± 3.4*	12.5 ± 3.1*	9.0 ± 3.1	11.7 ± 3.3
Basal estradiol (females only)	111.3 ± 15.2**	133.4 ± 18.3**	113.8 ± 13.9	119.1 ± 15.4
Uterine length, cm	4.10 ± 0.49*	4.59 ± 0.47*	4.00 ± 0.35	4.35 ± 0.37
Ovarian volume, cm^3^	2.94 ± 0.52*	3.43 ± 0.48*	2.86 ± 0.46	3.18 ± 0.46
Electronic device use (h)	1.6 ± 0.9***	3.9 ± 1.5***	–	–

* = *p* < 0.05; ** *P* = 0.005; *** = *p* < 0.0005. *BA* bone age, *CA* chronological age, *SDS* standard deviation score, *BMI* body mass index, *LH* luteinizing hormone, *FSH* follicle-stimulating hormone, *GnRH* gonadotropin releasing hormone test

Four patients were excluded from the study: two for CPP and cognitive delay (one of whom was adopted), one for a hypothalamic-pituitary malformation and one for puberty associated with Williams-Beuren syndrome.

**Fig. 1:**
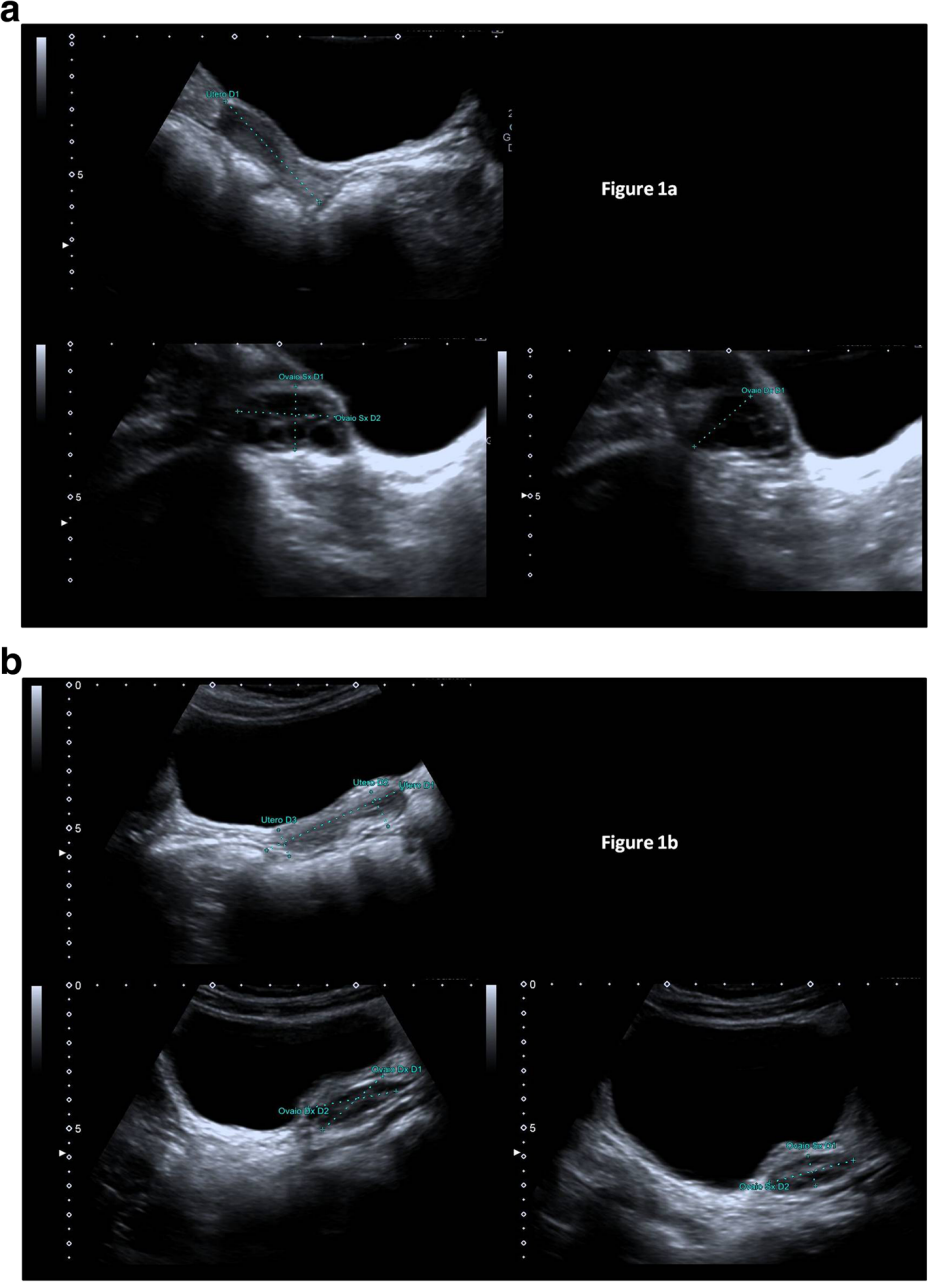
Two girls with precocious puberty diagnosed during lockdown for COVID-19. Patient 1 started puberty at 6.2 years (March 2020): May 2020: height 1.45 SDS, BMI 0.45 SDS; Tanner stage B3 PH2 AH1); patient’s pelvic sonography (May 2020; **a**). Patient 2 started puberty at 6.9 years (February 2020): May 2020: height 1.36 SDS, BMI 0.52 SDS; Tanner stage B3 PH1 AH1); patient’s pelvic sonography (May 2020; **b**)

**Fig. 2:**
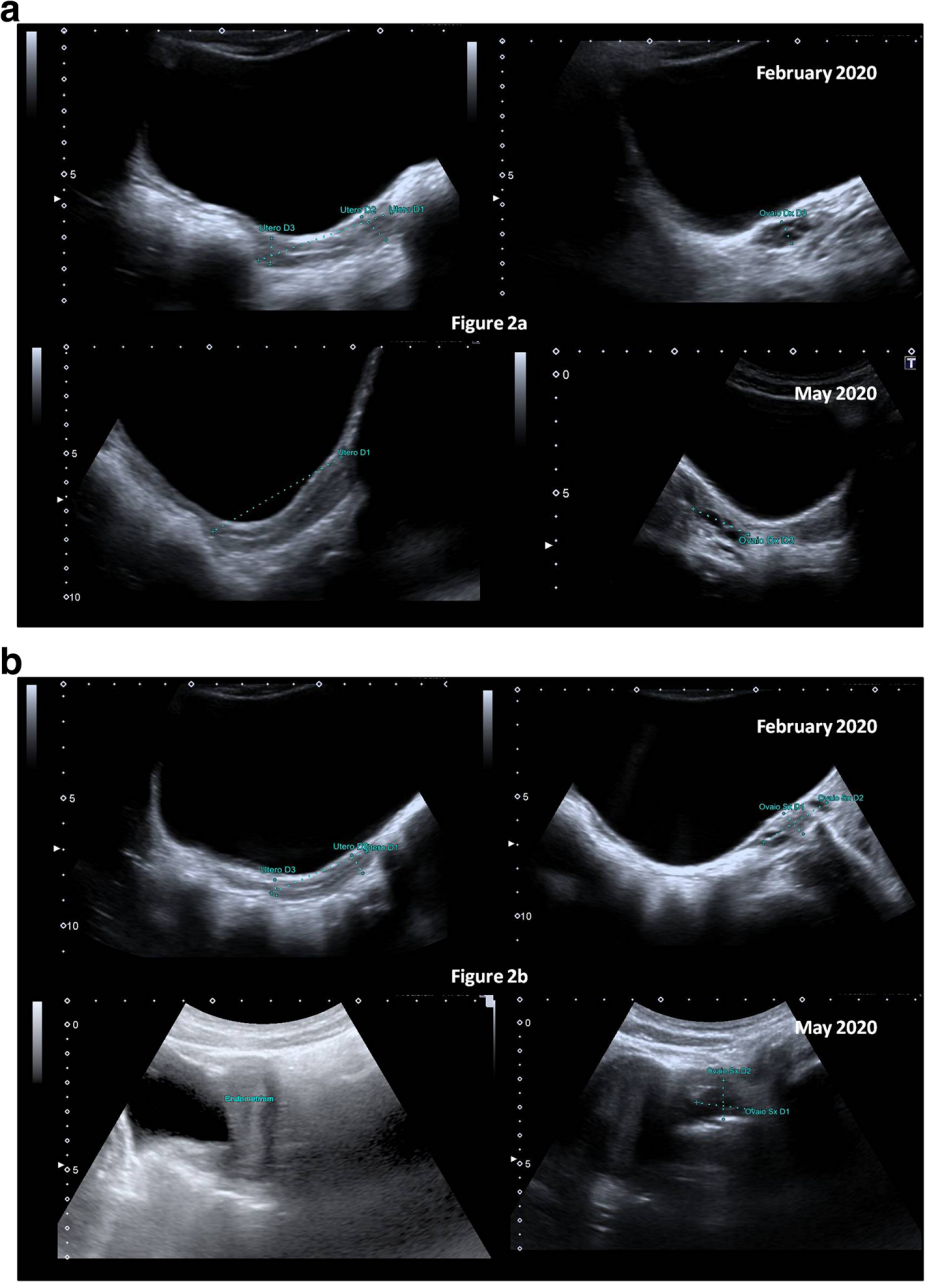
Two girls with central precocious puberty with a slow rate progression before lockdown and acceleration during or after lockdown for COVID-19. Patient 1 started puberty at 7.5 years (November 2019: height 1.23 SDS, BMI 0.88 SDS; Tanner stage B2 PH1 AH1); the second pelvic sonography, February 2020 (**a**) is similar to the sonography at diagnosis (uterine length 43 mm and ovary volumes respectively 1.8 and 1.2 mL); the sonography in May 2020 (**b**) showing an acceleration in the tempo of puberty (height 1.29 SDS, BMI 0.91 SDS; Tanner stage B3–4 PH2 AH1; uterine length 52 mm, ovaries volume 4,9 and 4,0 mL). Patient 2 started puberty at 7.4 years (October 2019: height 1.17 SDS, BMI 0.69 SDS; Tanner stage B2 PH1 AH1); the second pelvic sonography in February 2020 (**b**) is similar to the sonography at diagnosis (uterine length 45 mm and ovaries volume respectively 3.8 and 3.7 mL); the sonography in May 2020 shows also an acceleration in the tempo of puberty (height 1.22 SDS, BMI 0.76 SDS; Tanner stage B3–4 PH1 AH1; uterine length 63 mm and ovary volumes respectively 6.4 and 8.8 mL)

The overall number of new CPP diagnoses was significantly higher during and after lockdown for COVID-19 than the mean number of cases for the same period each year from 2015 to 2019 (*p* < 0.0005, Table 1 and Fig. 1). The number of patients with slow progression CPP who transitioned to fast progression CPP during and after the lockdown was also significantly higher (*p* < 0.0005, Table 2 and Fig. 2).

Compared with control group 1, the girls in group 1 of this study presented an earlier chronological age at B2 (*p* < 0.005), an earlier chronological age at diagnosis (*p* < 0.0005), and a more advanced Tanner stage at diagnosis (*p* < 0.05). However, there was no significant difference between the time between the appearance of the B2 and diagnosis in group 1 patients and controls (respectively 3.1 ± 0.9 vs. 3.0 ± 0.8 months).

The patients in group 1 also had more markedly elevated basal LH and E2 levels and a higher LH peak after LHRH test in comparison with the control group (*p* < 0.005). They also presented greater increases in uterine length and ovarian volume (*p* < 0.0005). Auxological parameters such as height SDS, BMI SDS and bone age SDS were not statistically different between group 1 patients and controls, but BMI SDS increased significantly in group 1 during the lockdown (*p* < 0.05).

Compared with the previous 5 years, during lockdown, the number of patients with CCP who experienced a transition from slowly progressive pubertal development to accelerated pubertal development was statistically significant (*p* < 0.0005). Compared with controls, patients in group 2 at the same stage in follow-up (0.31 ± 0.02 vs. 0.38 ± 0.02 years), had significantly elevated LH (*p* < 0.05) and E2 levels (*p* < 0.005), as well as a higher LH peak after LHRH test (*p* < 0.05) and a significantly accelerated progression rate as measured by the Tanner scale (*p* < 0.0005), uterine length (*p* < 0.05), and ovarian volume (*p* < 0.05). As in group 1, for patients in group 2, auxological parameters (height SDS, bone age SDS) were not statistically different from controls but Δ BMI SDS increased significantly (*p* < 0.005).

The evaluation of the use of electronic devices before and during the lockdown in the two groups, showed a significantly increased use (before: 1.6 ± 0.9 h/day; during: 3.9 ± 1.5 h/day, *p* < 0.0005). The main use was for school lessons and study (1.8 ± 0.8 h/day), but also for entertainment (TV and videogames) (1.3 ± 0.8 h/day vs. 0.7 ± 0.5 h/day, *p* < 0.0005). Use of devices during the hours before sleeping also significantly increased (*p *< 0.0005).

## Discussion

Our study shows a significantly increased incidence of new diagnoses of central precocious puberty and a faster rate of pubertal progression in previously diagnosed patients during and after the lockdown for COVID-19 in comparison with the same period of the 5 previous years. Our results seem to suggest a correlation between environmental factors and early onset and fast progression puberty. Our study focuses on female patients, but we hypothesize that such factors may also affect pubertal development in boys. As diagnosis in males is more frequent at the G2 stage of genital development, it would be interesting to evaluate any increases in the frequency of CPP in male patients over the coming months.

In humans, 70–80% of variances in pubertal timing and *tempo* can be explained by genetic factors, but the role of environmental factors is also well known [2, 5]. A number of studies, reported an earlier menarcheal age in girls with a low birth weight or in utero growth retardation, showing that the possible effects of the environment start during intrauterine life [26], although other studies contradict this conclusion [27].

Our study may help shed light on 3 factors hypothesized in the literature as potentially contributing to the timing and/or *tempo* of pubertal development: an increased BMI [28, 29], the “overuse” of electronic devices [21] and psychological triggers [30].

Nutrition plays a key role in the timing of puberty and could explain, at least partially, the secular trend of earlier development [31]. Obesity is associated with early menarcheal age [29, 32]. Leptin, insulin-like growth factor-I and glucose have been shown to be involved in the control of GnRH secretion, but their role in the timing of puberty remains controversial [31]. The effects of fat mass may also interact with the effect of some endocrine disrupters that have a high affinity for lipids and are stored in fat tissue, thus creating conditions for the persistence of systemic effects [31].

Psychological factors may also be important. It is likely that the COVID-19 pandemic impacted the mental health and well-being of children considerably. Illness, anxiety about becoming ill as well as prolonged social distancing may have lasting effects on children [33]. The closure of schools, the sudden disruption of extended social and family relationships, the change in daily habits and parental anxiety about financial and other problems may have affected children’s emotional stability and sense of security [33]. As yet there is little data on how children in all countries responded psychologically to the crisis but [34] the importance of psychological factors in precocious puberty has been reported in children migrating from developing to developed countries, primarily through international adoption [35]. It has been hypothesised that for adopted children who move from a deprived environment to a resource rich environment, environmental factors which slow or delay pubertal development are deactivated while factors which favour pubertal development are enhanced, triggering the onset of puberty [35]. Genetic factors cannot fully explain the high incidence of precocious puberty in internationally adopted girls as their average menarcheal age is lower than the average menarcheal age both in their foster countries and in their countries of origin [35].

Our data show an increased use of electronic devices; the time spent by the children on electronic devices increased by a factor of 2.5 during lockdown. We may hypothesize that the overuse of electronic devices contributes to the timing and/or *tempo* of puberty. A number of studies have recently investigated the effects of exposure to electromagnetic fields on melatonin [21, 36–38]. Exposure to simulated electromagnetic fields has been associated with decreased melatonin production by isolated pinealectocytes in vitro, as well as with a decreased pineal and plasma melatonin and its urinary metabolites [36]. The study in the town of Cavriglia showed an overall and age-dependent decrease in urinary melatonin concentration associated with children’s exposure to a television screen which was more pronounced in younger children [21]. Night-time serum melatonin levels are highest in very young children and drop progressively by 80% throughout childhood until adolescence and young adulthood [39]. There is some evidence that the drop in nocturnal melatonin levels parallels sexual maturation processes [40], as measured by the Tanner scale [41]. Animal studies also show that a reduction in melatonin may accelerate pubertal development [42] and that exogenous melatonin can suppress GnRH secretion [43]. It is plausible that a greater use of electronic devices leads to a reduction in melatonin levels which in turn triggers the endocrine changes that lead to the onset of pubertal development at an earlier age [44].

We are aware that this study has several limitations, including its small sample size. The COVID-19 pandemic caused profound and sudden changes in community care and hospital settings, making it impossible to create a homogeneous control group. We underline the need for more studies involving larger numbers of CPP patients. Moreover, our study did not assess decreases the changes in physical activity or in calorie intake in these children. However, increases in calorie intake and decreases in physical activity have been observed in the Italian population during lockdown [13] and it is likely that also our children were affected in a similar way.

## Conclusion

In conclusion, our data shows an increase in the incidence of new CPP diagnoses, as well as a faster rate of pubertal progression in previously diagnosed patients, during and after lockdown for COVID-19, suggesting that environmental factors, such as the BMI and the use of electronic devices, which emerged during the lockdown played a triggering role. Further studies are needed to confirm our data and elucidate the relative importance of the various factors at play and how they interact. In this uncertain period, clinicians should be aware of the signs of pubertal developmental disorders and should carefully monitor existing patients with CPP for signs of accelerated pubertal progression.

## Data Availability

Yes.
